# Supplementary material for: Changing attitudes towards female genital mutilation. From conflicts of loyalty to reconciliation with self and the community: The role of emotion regulation
Source: PLoS One. 2022 Jun 21;17(6):e0270088. doi: 10.1371/journal.pone.0270088 (PMC9212168; doi:10.1371/journal.pone.0270088)
Supplement: S1 Appendix — (DOCX) [file pone.0270088.s001.docx]

Completed COREQ Checklist_Plos

| No | Item | Guide Q/ description |
| --- | --- | --- |
| **Domain 1**  **Research team and**  **reflexivity** |  |  |
| **Personal**  **characteristic** |  |  |
| 1. | Interviewer/facilitator | The first author conducted the interviews |
| 2. | Credentials | BSc, MSc |
| 3. | Occupation | PhD student, Registered Nurse |
| 4. | Gender | Female |
| 5. | Experience and training | Registered Nurse, PhD Study in Public health |
| **Relationship with**  **participants** |  |  |
| 6. | Relationship established | Yes, through gatekeepers and snowball procedures. |
| 7. | Participant knowledge of the interviewer | No prior knowledge of the participants except the expert Kadyatou Diallo, the president of **GAMS** *(Groupe pour l’Abolition des Mutilations Sexuelle)* who later became involved in the theorisation process. |
| 8. | Interviewer characteristics | The interviewer is an African woman, a PhD student who is also the first author, and a health professional (nursing background). |
| **Domain 2**  **Study design** |  |  |
| **Theoretical**  **Framework** |  |  |
| 9. | Methodological orientation and Theory | A qualitative methodology informed by the narrative biographical approach and grounded theory approach was used to allow the theorisation process. Page 5, lines 124-130. |
| **Participant selection** |  |  |
| 10. | Sampling | 3 data sources: see page 6-7, lines 131-170  -the 15 women from the previous study were interviewed twice recruited through gatekeepers and snowball procedures.  - 10 books: the life stories and public testimonies of 10 women referred to as ‘norm leaders’ through websites and contact with organisation very active in the field.  - 6 experts: chosen for their complementary fields of expertise. Page 7 |
| 11. | Method of approach | -the 15 women were recruited through gatekeepers and snowball procedures.  - 10 books: the life stories and public testimonies of 10 women referred to as ‘norm leaders’ through websites and contact with the organisation very active in the field.  - 6 experts: personally approached for their theoretical expertise with regards to the emerging hypotheses and with no prior knowledge of FGM for 4 of them. One expert with lived experience of FGM, and one with clinical experience in relation to FGM. They were recruited in the University (UCLouvain, Belgium) and the one expert with lived experience is the founder and president of GAMS and was recruited in the organisation. Page 7,lines 131-170. |
| 12. | Sample size | -30 transcripts from 15 women interviewed twice.(previous study 1)  -10 books Page 6, line 138  -6 experts Page 6 |
| 13. | Non-participation | N/A |
| **Setting** |  |  |
| 14. | Setting of data collection | -15 women were interviewed either at GAMS, their homes, or in the office of the first author according to their preferences (Agboli et al., 2020) (Agboli A.A., Richard F., Aujoulat I. ‘When my mother called me to say that the time of cutting had arrived, I just escaped to Belgium with my daughter’: identifying turning points in the change of attitudes towards the practice of female genital mutilation among migrant women in Belgium. BMC Women’s Health. 2020;20: 107).  -the books  -The experts were interviewed via videoconference through Teams by the first and last authors. The consultation with the lived experience expert was done face-to-face by the first author alone. Page 6-7, lines 131-170. |
| 15. | Presence of non-participants | N/A |
| 16. | Description of sample | -The sample of the 15 women is described in the article (Agboli A.A., Richard F., Aujoulat I. ‘When my mother called me to say that the time of cutting had arrived, I just escaped to Belgium with my daughter’: identifying turning points in the change of attitudes towards the practice of female genital mutilation among migrant women in Belgium. BMC Women’s Health. 2020;20:107).  -the books, see page 6-7.  -The experts: Page 6-7 |
| Data collection |  |  |
| 17. | Interview guide | -The interview guide of the 15 women is in article (Agboli et al., 2020).  -The interviews of the experts were based on emerging hypotheses from our study 1 (Agboli *et al.,* 2020) |
| 18. | Repeat interviews | Repeat interviews of the 15 women. See article (Agboli *et al.,* 2020) |
| 19. | Audio/ visual recording | Audio recording: The women’s stories were recorded, and their consent was asked for beforehand in the previous study.(see Agboli et al., 2020). |
| 20. | Field notes | Yes. Extensive notes were taken during the interviews with the experts and we sent the synthesis which they had the opportunity to read, revise and validate. Page 7, lines 159-167. |
| 21. | Duration | -The 15 women: see Agboli et al., 2020  -Consultation with experts: between 45 and 1h. The duration of the face-to-face interview was 45 min.  Page 7, lines 156-167. |
| 22. | Data saturation | Our emerging theorisation would deserve further testing through theoretical sampling to ascertain saturation. Page 28 lines 698-711.  The multiplicity of data sources: the original interviews, the books analysis and the consultation with the experts tend towards saturation even though we are aware that our model could be further tested through theoretical sampling. Page 28-29 lines 684-709. |
| 23. | Transcripts returned | Yes.  -the 15 women: had a chance to look at the lifelines and co-constructed the turning points with us, confirmed them, and validated them (see Agboli et al. 2020).  -The experts: synthesis of notes taken returned to them and validated them. Page 7, lines 163-167. |
| **Domain 3**  **Analysis and findings** |  |  |
| **Data analysis** |  |  |
| 24. | Number of data coders | Up to three:  -The 15 women: Three researchers (AA, IA, FR) were involved in the analysis and interpretation of the 30 transcripts.  -The analysis of the books: 2^nd^ author (FR) and the first author (AA).  -The experts: Analysis and theoretical integration of the experts’ contributions were done by the first author (AA) and the last author (IA).  See page 10-11, lines 238-268. |
| 25. | Description of the coding tree | Yes, the authors provided a description of the initial model of emotion regulation and the conflict of loyalty. |
| 26. | Derivation of themes | We started analysing the data using a framework analysis approach based on the hypotheses generated from the previous study (Agboli et al., 2020) and proceeded inductively with predefined categories, and developed our model of change. See Page 11, lines 261-268. |
| 27. | Software | We used Excel software to group the codes. |
| 28. | Participant checking | Yes.  -The 15 women: the second interview and co-construction of turning points (Agboli et al., 2020)  -The experts: revision and validation of the synthesis of their interviews sent to them and later, the discussion, interpretation, and validation of the final model. Page 11; lines 246-268. |
| **Reporting** |  |  |
| 29. | Quotations presented | Yes, pages 12-22. |
| 30. | Data and findings consistent | Yes, pages 12-22 |
| 31. | Clarity of major themes | Yes, page 12-22. |
| 32. | Clarity of minor themes | N/A |
