# Supplementary material for: Changing attitudes towards female genital mutilation. From conflicts of loyalty to reconciliation with self and the community: The role of emotion regulation
Source: PLoS One. 2022 Jun 21;17(6):e0270088. doi: 10.1371/journal.pone.0270088 (PMC9212168; doi:10.1371/journal.pone.0270088)

**Supporting information 2:** A fictive vignette of a typical reconstructed story from different participants after the 1st and 2nd interviews*. (Agboli et al., 2020)*

*All names and some identifying details have been changed to protect the anonymity and confidentiality of the migrant women*.

**1^st^ interview**: Sarah (fictive name) was 42 years old at the time of the interview and came from a high prevalence FGM-practicing country. She has been living in Belgium for nine years since her refugee status has been granted. She has two girls and two boys all born in her home country and fled from there because her mother-in-law put pressure on her and had arranged for the girls to undergo the practice of FGM. She remembered her own experience of the procedure and felt the pain for her daughters.

She had to hide and contacted a smuggler who helped her through until she got into the country.

Her stay in the asylum seekers centre after her arrival in Belgium made her to be confronted with the noise of urine in the bathrooms from other women from other ethnic backgrounds. She realised she was different, and she cried.

Her own experience of FGM was back to when she was eight years old, and she underwent FGM at the request of her grandmother who told her that she would become clean and important in the community. The feeling afterwards was extreme pain, hatred and loss of something instead of gaining something even though she received lots of presents. She suffered a lot of period pain during her teenage years. She went through forced marriage and described the sexual intercourse as nightmares as it was very painful to the point she does not have any desire for it at such. Her child delivery came along with painful labour too, and she understood the link with FGM.

**2^nd^ interview**: Sarah expanded more about her sexual experiences for not being fulfilled. She saw it as part of herself as a woman, and mentioned that she was taught otherwise to endure pain as part of her fate, but thought she was entitled to pleasure and desire, not pain. She also reported the urgency she felt to protect her daughters and reflected on her own trauma experiences. Thus, she wanted to be a good mother for them. So, she took action to flee from the country and later became an activist to fight and protect her daughters until they become adults.

Lifeline for the 1^st^ interview with few events: E represents events


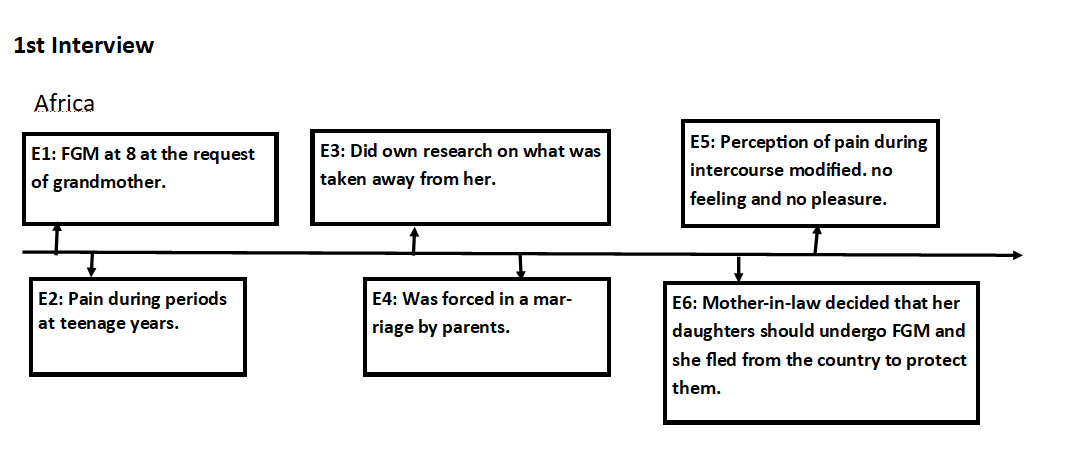


2^nd^ interview: More events - confirmation of TP- new TP

TP in the home country (confirmation of TP)

TP in Belgium (new TP)


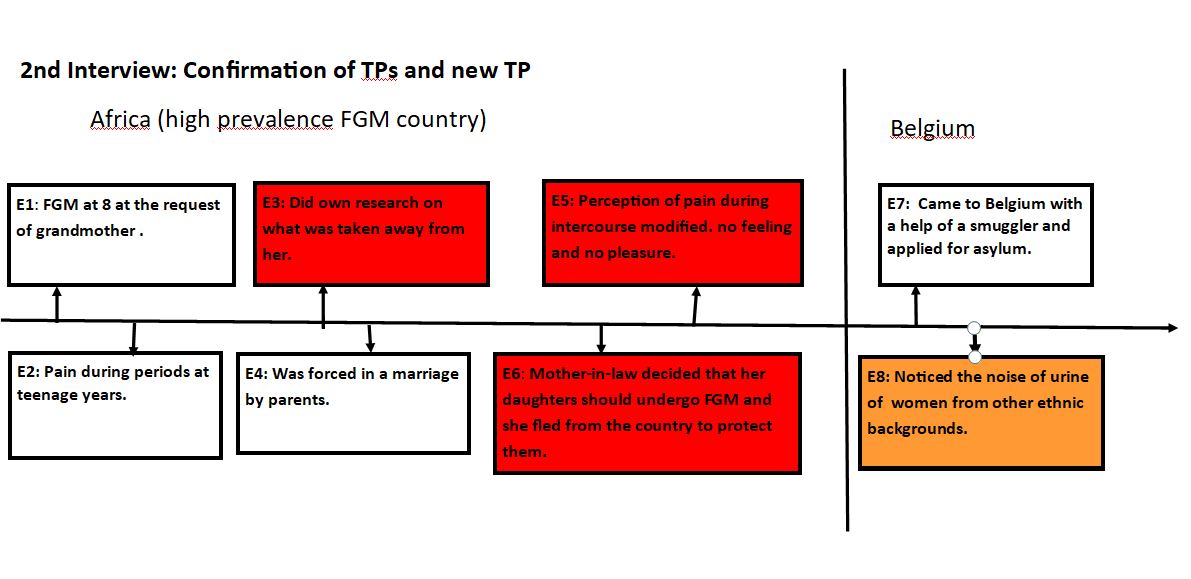

Supplement: S2 Appendix — (Agboli et al., 2020). (DOCX) [file pone.0270088.s002.docx]
