# Supplementary material for: Changing attitudes towards female genital mutilation. From conflicts of loyalty to reconciliation with self and the community: The role of emotion regulation
Source: PLoS One. 2022 Jun 21;17(6):e0270088. doi: 10.1371/journal.pone.0270088 (PMC9212168; doi:10.1371/journal.pone.0270088)
Supplement: S3 Appendix — (DOCX) [file pone.0270088.s003.docx]

**Interview guide** *(Agboli et al, 2020)*

*Thank you for your time and participation in this interview. It should last about 1 hour. The aim of the interview is to identify in the life stories the significant events that influenced the change of attitudes towards the practice of FGM.*

**First interview**

Could you tell me about your life experiences, and in doing so, include any story in your life that you think is important? You can start wherever you want.

**Second interview**

*The purpose of the second interview is for the women to confirm the hypotheses of turning points, to narrate more events, to identify other relevant turning points, and to complete the lifelines together with the women. The second interview was guided by semi-structured questions that were unique to each woman according to their initial narratives.*

-Please could you confirm the hypotheses of *turning points* raised after the first interview?

-Could you tell me more of any event you think that has contributed to any awareness towards the change?

Based on each woman’s life story

- What led you to question the excision?

-When you were growing up, what do you think the reason for the cutting was?

-How do you position yourself regarding FGM now?

-How do you feel about it now?
