# Supplementary material for: Changing attitudes towards female genital mutilation. From conflicts of loyalty to reconciliation with self and the community: The role of emotion regulation
Source: PLoS One. 2022 Jun 21;17(6):e0270088. doi: 10.1371/journal.pone.0270088 (PMC9212168; doi:10.1371/journal.pone.0270088)
Supplement: Attachment — Submitted filename: Response to Reviewers.docx [file pone.0270088.s005.docx]

To the manuscript Editor: Dr Forough Mortazavi

Academic Editor

Plos One	Afi Agboli

UCLouvain/IRSS

30, Clos Chapelle-aux-champs

1200 Brussels

Brussels, 09th May 2022

Dear Dr Mortazavi,

Please find attached for your kind review our revised new manuscript and title for consideration by *PLOS ONE.*

The title is: ***Changing attitudes towards female genital mutilation. From conflicts of loyalty to reconciliation with self and the community: The role of emotion regulation***

We would like to thank you for reviewing this article. We have revised the manuscript taking into account the different comments from the reviewers and also from the editors. We added one reference as requested by Reviewer 2.

We confirm that this work is original and has not been published elsewhere, nor is it currently under consideration for publication elsewhere.

In this paper, we hypothesised that emotion regulation and the resolution of conflicts of loyalty were possible mechanisms that explain the change of attitudes towards the practice of female genital mutilation (FGM).

We believe that our emerging theorisation and the proposed model contribute to the body of knowledge and pave avenues to be explored by health professionals (who work with migrant women with FGM) when they develop a strategic intervention for the prevention of the practice of FGM.

We do not have any competing interests and we confirm that all authors have approved the manuscript and agree with its submission to *PLOS ONE.*

We believe that this manuscript is appropriate for publication by *PLOS ONE* because it gives information to students, academics, and organisations that promote women’s health.

Please address all correspondence concerning this manuscript to us at:

[afi.agboli@uclouvain.be](mailto:afi.agboli@uclouvain.be)

afisophieagboli@gmail.com

Thank you for your consideration of this manuscript.

Yours sincerely,

Afi Agboli (PhD student).

PS: Please see below the answers to the comments of reviewers.

**Revision 2**

**Reviewer #2: Congratulation. The manuscript has improved significantly. I only have a couple of comments for improvements.**

-In the method section review the next phrase: A qualitative methodology informed by the grounded theory approach was used to allow us and 125 it allowed us to generate the hypotheses mentioned above [8].

Answer: Thank you very much for this comment. We have now rearranged the sentence. Please see Page 5 lines 124-125 in the main manuscript.

A qualitative methodology informed by the grounded theory approach was used to allow us to generate the hypotheses mentioned above [8].

- It could be confusing to readers that there are 30 transcripts from 15 participants. At some point, it should be explained that 2 interviews were conducted with each participant and the reason.

Answer: Thank you very much for this comment. We have corrected it. Please see page 6, line 134-144 of the main manuscript.

(a) Fifteen women were interviewed twice in our previous study [8]. We chose the method of biographical narrative interviewing method (BNIM) as developed by Wengraf [35] in order to produce narratives relating to life events. In the BNIM approach to data collection, the interviewee is seen in two phases and sometimes three (not always present), with the first interview being unstructured and the consecutive interviews building on the previously collected data [35]. Thus, we had 30 transcripts derived from the interviews of the 15 women participants in this study who had all undergone FGM in their countries of origin during childhood. They all originated from sub-Saharan African countries and were living in Belgium at the time of the interview. Full details of the sample and the description of the process of the interviews can be found in our publication [8].

- In data source section:

As in the first data source is detailed. It should also be detailed in the third data source that the transcripts of the interviews, field notes... are the data to be analyzed.

Answer: Thank you very much for the comment. We highlighted it on page 11, lines 257-260 in the main manuscript.

The notes taken during the different discussions were compared and analysed by the first and last authors to further clarify our emerging theorisation, which was also discussed with co-author FR on several occasions and enriched through a review of some published material suggested by the experts.
